# Exopolysaccharide from *Ganoderma applanatum* as a Promising Bioactive Compound with Cytostatic and Antibacterial Properties

**DOI:** 10.1155/2014/743812

**Published:** 2014-07-10

**Authors:** Monika Osińska-Jaroszuk, Magdalena Jaszek, Magdalena Mizerska-Dudka, Adriana Błachowicz, Tomasz Piotr Rejczak, Grzegorz Janusz, Jerzy Wydrych, Jolanta Polak, Anna Jarosz-Wilkołazka, Martyna Kandefer-Szerszeń

**Affiliations:** ^1^Department of Biochemistry, Maria Curie-Sklodowska University, Akademicka 19, 20-033 Lublin, Poland; ^2^Department of Virology and Immunology, Maria Curie-Sklodowska University, Akademicka 19, 20-033 Lublin, Poland; ^3^Department of Comparative Anatomy and Anthropology, Maria Curie-Sklodowska University, Akademicka 19, 20-033 Lublin, Poland

## Abstract

A new exopolysaccharide preparation isolated from stationary cultures of the white rot fungus *Ganoderma applanatum* (GpEPS) was tested in terms of its bioactive properties including its cytotoxic and immunostimulatory effect. The results indicate that the tested GpEPS (at concentrations above 22.85 *µ*g/mL and 228.5 *µ*g/mL) may exhibit selective activity against tumor cells (cell lines SiHa) and stimulate production of TNF-*α* THP-1-derived macrophages at the level of 752.17 pg/mL. The GpEPS showed antibacterial properties against *Staphyloccoccus aureus* and a toxic effect against *Vibrio fischeri* cells (82.8% cell damage). High cholesterol-binding capacity and triglycerides-binding capacity (57.9% and 41.6% after 24 h of incubation with the tested substances, resp.) were also detected for the investigated samples of GpEPS.

## 1. Introduction

Numerous fungal preparations are used in traditional Eastern medicine for prevention and treatment of diseases, such as migraine, hypertension, arthritis, bronchitis, asthma, diabetes, hypercholesterolemia, and hepatitis. Among many species, the genus* Ganoderma* seems to be the most interesting mainly due to its wide therapeutic effect [1]. According to the available research, biologically active substances obtained from* G. applanatum* can be used in cancer treatment; moreover, they show a therapeutic effect against HIV [1].

Recent explosion of interest in isolation and characterization of bioactive compounds with unique properties from family Ganodermaceae may be observed. Among them, polysaccharides, especially glucans, deserve special attention [2]. Polysaccharides include a large and diverse group of substances that play an important role in the structure and function of fungal cell walls, which is the main polysaccharide source. However, it should be mentioned that, depending on the culture conditions, some fungal species also effectively produce fractions extracellular polysaccharides.

One of the most frequently studied biological properties of fungal polysaccharides is their antitumor activity. The antitumor effect depends on their immunomodulatory activities affected by many physical and chemical properties such as the chemical composition of the molecule, the degree of branching, the type of glycosidic bonds, conformation, or molecular weight [3]. Among the number of fungal polysaccharides described, *β*-glucans containing mainly *β* (1 → 3)-glycosidic bonds and having side chains linked by *β* (1 → 6)-glycosidic bonds have been presented as the most active [4]. It is supposed that inhibition of tumor cell growth is the result of *β*-glucan-dependent stimulation of macrophages and dendritic cells followed by secretion of various cytokines including TNF-*α*, IFN-*γ*, and IL-1*β*, and stimulation of NK T and B cells [5, 6]. Another possible mechanism of the impact of *β*-glucans on immune cells is the interaction of these polysaccharides with the CR3 receptors [7, 8]. Besides their action on immune cells, *β*-glucans also inhibit angiogenesis by cutting off the supply of nutrients to tumor cells and, in consequence, inhibiting their development [9].

Redox processes in living organisms are the basis for obtaining energy necessary for the proper conduct of metabolic changes. However, uncontrolled production of highly reactive forms of free radical compounds can be a cause of damage to genetic material, initiation of carcinogenesis, and cell degradation associated with aging processes. To prevent radical-mediated disorders, many natural compounds exhibiting antioxidant properties can be used and polysaccharides are the main group of them. Some reports have indicated that the antioxidant properties of the intracellular polymers produced by* G. lucidum* and* G. applanatum* may be correlated with the content of polyphenolic compounds in the samples. Polyphenols have been described as powerful antioxidants due to their redox potential, which allows them to act as reducing agents and hydrogen donors as well as singlet oxygen scavengers [10–14].

There are many available reports describing antibacterial properties of fungal polysaccharides in relation to both gram-positive and gram-negative bacteria. For example, it has been discovered that the lentinan obtained from the fungus* Lentinus edodes* exhibits antibacterial properties. Hirasawa et al. [15] proved that substances from dried Shiitake mushrooms (*L. edodes*) showed efficient antibacterial activities against* Streptococcus* spp.,* Lactobacillus* spp.,* Actinomyces* spp.,* Porphyromonas* spp., and* Prevotella* spp. of oral origin.

The above findings suggest that exploration of the world of fungal extracellular polysaccharides seems to be a very interesting issue for medicinal application, given the ease of isolation and production thereof, compared with intracellular polysaccharide preparations. The aim of the present work was isolation of the extracellular polysaccharide (GpEPS) produced by stationary cultivated* G. applanatum* and characterization of its chemical composition, structure, and biological (antimicrobial, antitumor, immunostimulatory, and antioxidative) activities. Additionally, the cholesterol-binding capacity, triglyceride-binding capacity, and glucose-binding capacity of the GpEPS preparation were also analyzed.

## 2. Materials and Methods

### 2.1. Microorganism and Culture Conditions

The* G. applanatum* strain was obtained from the Fungal Collection (FCL) of the Biochemistry Department, Maria Curie-Sklodovska University, Lublin, Poland. The cultures were maintained on potato-dextrose-agar (PDA) plates, which were inoculated and incubated at 25°C for 7 days and stored at 4°C. The experimental inocula were prepared in 100 mL Elenmeyer flasks containing 25 mL of the PDA medium at 25°C for 7 days. After inoculation with 4% (v/v) of homogenate, rotary shaking cultures were incubated in 250 mL Erlenmeyer flasks containing 100 mL medium. The media consisted of the following components: 30 g/L glucose, 1 g/L (NH_4_)_2_SO_4_, 0.5 g/L KH_2_PO_4_, 0.5 g/L MgSO_4_ × 7H_2_O, 0.01 g/L FeSO_4_z7H_2_O, and 1 g/L yeast extract. The experiments were performed at 25°C in a rotary shaker (120 rpm) for 12 days. After this time, the culture liquid was separated from the mycelium by centrifugation for 15 min in 4°C at 10.000 rpm.

### 2.2. Genomic DNA Isolation and Amplification of ITS Sequences

A culture of* Ganoderma applanatum* was grown stationary in Lindeberg and Holm medium [16] at room temperature (25°C) for 7 days. Mycelia were harvested through Miracloth (Merck, Whitehouse Station, NJ, USA), washed twice with TE buffer, and frozen in liquid nitrogen. DNA was isolated according to Borges et al. [17]. The purity and quantity of the DNA samples were evaluated using an ND-1000 spectrophotometer (Thermo Scientific, West Palm Beach, FL, USA).

PCRs were performed using Sigma RedTaq in a Tpersonal thermal cycler (Biometra, Goettingen, Germany). To confirm the identity of the fungus, the ITS region in the nuclear ribosomal repeat unit was determined by direct sequencing of the PCR products amplified with ITS1-ITS4 primers as described previously [18].

### 2.3. Extraction of Exopolysaccharides

Crude exopolysaccharides in the culture liquid were precipitated with cold 96% ethanol in the ratio 1 : 4 (v/v) and kept overnight at 4°C. The resulting preparation was centrifuged (10 000 rpm, 10 min.), washed three times with ethanol, dissolved in distilled water, and lyophilized.

### 2.4. General Properties of Crude Exopolysaccharides

#### 2.4.1. FT-IR Spectroscopy Analysis

Complete acid hydrolysis of the exopolysaccharides was carried out with 4.95N trifluoroacetic acid (TFA) at 80°C in a heating block for 4 h, and next the mixture was cooled, evaporated, and then analyzed using infrared spectroscopy. The FT-IR spectra of the exopolysaccharides were recorded on a Thermo-Nicolet Model 8700 A spectrophotometer with a FT Ramana Nicolet NXR module (Thermo Scientific, USA). The spectra were recorded in a wavelength range of 4000–400 cm^−1^ using the KBr disc technique.

#### 2.4.2. Determination of Total Carbohydrate and Reducing Sugar

The total carbohydrate content of the exopolysaccharides was determined according to Dubois et al. [19] using the phenol-sulfuric acid assay with D-glucose as a standard. The concentration of reducing sugars was measured by the Somogyi-Nelson method based on the procedure described by Hope and Burns [20] with some modifications. The amount of total reducing sugars was calculated with D-glucose as a standard. The final total value of polysaccharides was obtained by subtraction of reducing sugars from the total carbohydrates.

#### 2.4.3. Determination of Proteins and Phenolic Compounds

The protein concentration was estimated by the Coomassie brilliant blue (G-250) dye-binding method [21] using Bio-Rad dye stock solution with bovine serum albumin (Sigma) as a standard. The total phenolic compounds content of GpEPS was determined with diazosulfanilamide by the DASA test [22]. The changes in absorbance were measured at 500 nm and compared with the standard curve of vanillic acid.

#### 2.4.4. Microscopic Imaging of Exopolysaccharide Using Confocal Laser Scanning Equipment

The visualization of GpEPS morphology was conducted according to the method described in the earlier report [23]. Fluorescence Brightener 28 was used for proper detection of *β*-linked polysaccharides. As presented in the earlier report, the lyophilized samples of extracted exopolysaccharides (1 mg) were washed with MQ water and after water removal they were stained for 30 min with 200 *μ*L of 25 *μ*g/mL Fluorescence Brightener 28. Then the sample was washed twice with water to remove the dye, placed on a glass slide and estimated under a microscope. An inverted microscope Axiovert 200M equipped with an LSM 5 Pascal head (with magnification 200x) was used for visualization of the GpEPS structure.

### 2.5. Biological Properties of Crude Exopolysaccharides

#### 2.5.1. DPPH Radical Scavenging Activity

Free radical scavenging activity of the crude exopolysaccharides was estimated by the 1,1-diphenyl-2-picrylhydrazyl (DPPH^.^) assay, described by Paduch et al. [24]. The tested compound (0.1 mL) at concentrations ranging from 6.25 to 800 *μ*g/mL was added to 0.1 mL of DPPH^.^ solution (0.2 mg/mL in ethanol). Trolox standards well known for their strong antioxidant activity were used as a positive control. Absorbance at 515 nm was estimated after 2, 5, 10, 15, 20, and 30 min. of incubation at room temperature. The capability of scavenging DPPH^.^ radicals was calculated by the following formula:

(1) $$\begin{array}{c} {\text{DPPH}}^{.}\;\text{scavenging}\;\text{effect}\;\left(\%\right)=\left[\frac{X_{0}-X_{1}}{X_{0}}\right]\times 100, \end{array}$$

where *X*_0_ is the absorbance of the control and *X*_1_ is the absorbance of the tested compound/standard. The inhibition curves were prepared and EC_50_ values were obtained as described previously [23].

#### 2.5.2. Estimation of the Toxicity Effect Using the Microtox Protocol

The toxic effect of the tested EPS from* G. applanatum *cultures towards marine bacterium* Vibrio fischeri* was estimated using the Microtox Model 500 Analyzer detection system according to the procedure described in the earlier report [23]. The toxicity test used in the present report is based on the study of luminescence intensity of genetically modified bacteria. Any changes of cell respiration are closely correlated with the cellular activity and they cause a reduction of luminescence. The intensity of light of* V*.* fischeri* cells was measured at 0, 5, and 15 min after the treatment with the GpEPS fraction. The research method applied was conducted according to the Screening Test Protocol of the Microtox assay.

#### 2.5.3. Cytotoxic Activity of GpEPS

Cervical carcinoma cell lines SiHa (ATCC, HTB-350) and Ca Ski (ATCC, CRL 1550) were used to determine the antitumor activity of this preparation. The SiHa cell line was established from squamous cell carcinoma (primary tumor), containing HPV serotype 16. This cell line was maintained in MEM supplemented with 10% fetal bovine serum (FCS). The Ca Ski cell line was established from epidermoid carcinoma derived from a metastatic site in the small intestine. The cells contained HPV serotypes 16 and 18. This cell line was maintained in RPMI 1640 supplemented with 10% fetal bovine serum (FCS). In this study, a human skin fibroblast (HSF) cell line was used as a model of normal cells. The HSF cell line was established from skin explant and maintained in DMEM/MEM (1 : 1) supplemented with 10% FCS.

*(1) MTT Assay*. The MTT assay is a colorimetric cytotoxicity and proliferation detection assay, based on the metabolic activity of viable cells. Tetrazolium salts (MTT) are reduced only by metabolically active cells, namely, by a mitochondrial enzyme, to a blue colored fromazan, whose amount is proportional to the number of viable cells.* Cytotoxicity assay. *The SiHa and Ca Ski cell lines (5 × 10^5^ cells/mL) and HSF (1 × 10^5^ cells/mL) were seeded in a 96-well microtiter plate and cultivated under standard conditions (5% CO_2_ at 37°C) for 24 hours. In the case of the immunomodulatory activity assay, the cytotoxicity of the tested fraction was determined toward THP-1-derived macrophages. The culture medium was discarded from the wells and the cells were incubated for 24 and 48 hours with various concentrations of fraction samples as indicated in the figure. The fraction samples were prepared using a medium with 1% FCS appropriate for the cell line. Cells in the medium with 1% FCS alone were used as a positive control. After incubation, the MTT solution (final concentration 1 mg/mL) was added to each well and incubated for 3 hours. Next, 100 *μ*L of the SDS solution was added to each well to dissolve formazan crystals. The plates were incubated for 24 hours at 37°C. The optical density was measured on a microtitre plate reader (Bio-Tek Instruments, Inc.) at 570 nm. The cytotoxicity of the tested fraction was determined from absorbance values and expressed as percentage relative to the control (100% of living cells).* Proliferation assay*. SiHa and Ca Ski cell lines (5 × 10^4^ cells/mL) and HSF (3 × 10^4^ cells/mL) were seeded in a 96-well microtiter plate and cultivated under standard conditions (5% CO_2_ at 37°C) for 24 hours. Afterwards, the medium was discarded and the cells were incubated for 96 hours with various concentrations of fraction samples as indicated in the figure. The fraction samples were prepared using a medium with 10% FCS appropriate for the cell line. Cells in the medium with 10% FCS alone were used as a positive control. The MTT assay was carried out as described above (the cytotoxicity assay).

#### 2.5.4. Immunomodulatory Activity

Monocytic cell lines of varying degrees of differentiation are frequently used as a macrophage model. In this case, THP-1, an acute monocytic leukemia cell line (ATCC) was used to determine the immunomodulatory activity of the* G. applanatum *GpEPS fraction. Before the assay, the THP-1 cells were treated with phorbol-12-myristate-13-acetate (PMA). PMA treatment, which activates protein kinase C (PKC), induces differentiation of THP-1 cells into macrophages.

*(1) THP-1 Cell Differentiation*. The THP-1 cell line was maintained in RPMI 1640 supplemented with 10% fetal bovine serum (FCS) and 2 mM/L L-glutamine. The THP-1 cells (5 × 10^5^ cells/mL) were differentiated using 50 ng/mL of PMA for 3 days in 5% CO_2_ at 37°C. Afterwards, the PMA-containing medium was discarded and adherent cells were gently washed three times with RPMI 1640 (without FCS). Next, THP-1 derived macrophages were cultivated in RPMI (10% fetal bovine serum (FCS) and 2 mM/L of L-glutamine) for three days with daily changes of the medium. The macrophages cell cultures obtained were used to determine the immunomodulatory activity of the* G. applanatum *GpEPS fraction, which was followed by determination of the cytotoxicity (MTT assay) of the tested fraction (described above).

*(2) Immunomodulatory Activity Assay*. The immunomodulatory activity of the* G. applanatum *exocellular polysaccharide fraction was determined using THP-1-derived macrophages that were able to synthesize and secrete IL-6 and TNF-*α*. The level of cytokines was measured using the ELISA method (BD OptEIA, BD Biosciences) in cell culture supernatants of macrophages treated with a noncytotoxic concentration of the tested fraction. The THP-1-derived macrophages cultivated in RPMI with 2% FCS were the negative control, whereas cells treated with LPS of* E. coli*, serotype 0111 : B4 (10 *μ*g/mL), constituted the positive control. The cell cultures were incubated for 6 and 24 hours in 5% CO_2_ at 37°C. After incubation, cell culture supernatants were collected and centrifuged for 5 min, (14000 rpm) at 4°C. The samples were stored at −80°C until the ELISA assay. The ELISA assay was carried out according to manufacturer's instruction. The IL-6 level was determined after 24 hours, whereas TNF-*α* after 6 and 24 hours of incubation.

#### 2.5.5. Analysis of Antibacterial Activity

Antibacterial activity of the GpEPS fractions was tested using reference bacterial strains* Escherichia coli* (ATCC 25922) and* Staphylococcus aureus* (ATCC 25923).* E. coli* and* S. aureus* inocula (1° in McFarland scale) were kept under sterile conditions on Mueller-Hinton Agar II (Lab M, IDG plc, UK) (on Petri dishes). The isolated exopolysaccharide fractions (1 mg/mL) were applied to these agar plates in an amount of 100 *μ*L per well. The plates were incubated for 2 h at room temperature and then for 18 h at 37°C. Subsequently, the* E. coli* and* S. aureus* inhibition zones were measured. The minimum inhibitory concentration (MIC) of the GpEPS fractions obtained was measured according to the recommendations of the National Committee for Clinical Laboratory Standards.

#### 2.5.6. Testing the Ability of Exopolysaccharides to Bind Cholesterol, Triglycerides, Glucose, and Magnesium and Iron Ions

Standard human serum containing appropriate test substances (cholesterol, triglycerides, glucose, and magnesium and iron ions) were mixed with exopolysaccharides (1 mg/mL) in a proportion of 1.5 : 0.5 (v/v). The final concentrations of the test substances in the human serum were as follows: cholesterol 192 mg/dL, triglycerides 109 mg/dL, glucose 147 mg/dL, magnesium ions 2.86 mg/dL, and iron ions 174 *μ*g/dL. The samples were incubated for 2 and 24 hours at room temperature. After this time, the samples were centrifuged and assayed towards appropriate biochemical parameters. The comparative control was a sample of human serum containing distilled water instead of GpEPS. The concentration of plasma triglycerides, cholesterol, glucose, and magnesium, and iron ions was evaluated by commercially available biochemical test kits (Alpha Diagnostics, Poland).

### 2.6. Statistical Analysis

All the results are expressed as mean ± SD from three experiments (*n* = 3). Data were analyzed using one-way ANOVA followed by a post hoc Tukey's test. Values of *P* ≤ 0.05 were only reported as statistically significant.

## 3. Results and Discussion

Fungal species belonging to genus* Ganoderma* are known for their ability to produce a number of substances with promising biomedical properties. The fruiting bodies of* G. applanatum* are very often used in traditional Chinese medicinal therapies. They are known as very efficient anticancer, immunostimulatory, and antiviral factors [25, 26]. Hitherto, many papers have been published indicating that* G. applanatum* mycelia comprise certain amounts of saponins, flavonoids, cordial glycosides, steroids, and polysaccharides [26, 27]. A particularly interesting and still poorly studied group of compounds is extracellular polysaccharides extracted from* G. applanatum*.

### 3.1. PCR Amplification of the ITS Region

The strain of* G. applanatum* used in this study was genetically identified by determination of ITS sequences. One product of 654 bp was obtained from PCR with ITS1-ITS4 primers and followed by direct sequencing. The complete sequences of this product indicated over 99% identity to the* G. applanatum* ITS sequences and was deposited in GenBank under accession number JN008873.

### 3.2. General Properties of Crude GpEPS Preparation

In the present work, 12-day-old rotary shaken cultivated cultures of* G. applanatum* were used in order to obtain culture fluid for extraction of exopolysaccharides. Similarly, in their study, Lee et al. [26] showed that the highest production of exopolysaccharides was obtained from 12-day-old culture of* G. applanatum*. Currently, some fungal polysaccharides are obtained from fruiting bodies by means of time-consuming, multistep procedures for isolation and fractionation consisting in sugar ethanol precipitation, repeated extraction with boiling water and ammonium oxalate solutions of NaOH. The extracted polysaccharides are then purified by a variety of steps of chromatographic techniques. In this work, the preparation of extracellular polysaccharides was obtained by a simple ethanol precipitation procedure from the culture liquid of* G. applanatum*. Our initial experiments proved that the new extraction method with a yield of about 8.13% (Table 1) might be the most efficient method for isolation of exopolysaccharides from different strains of family Ganodermaceae. In contrast, Zhao et al. [28] received four times lower extraction efficiency (2.07%) for the crude polysaccharide obtained from* Ganoderma lucidum*.

The total carbohydrate content of the exopolysaccharide extracts from* G. applanatum* was 303 mg/g dcw (dry weight of crude polysaccharide) of the extract (30.3%). The amounts obtained in the present work are higher than the quantities of crude hot water extracted polysaccharides yielded by* G. applanatum *and* G. lucidum*, which were 20% and 30%, respectively [13]. Telles et al. [29] found 22.6% total carbohydrate in native extracellular polysaccharides from* Pleurotus sajor-caju*. However,* Cordyceps sinensis *was shown to comprise from 46% to 70% of total sugar, depending on the culture day [30]. The total polysaccharide content of the presented exopolysaccharides (241.8 mg/g dcw) (Table 1) was significantly higher than those reported for crude hot water extracted polysaccharides from* G. lucidum* (27.6 mg/g),* Agaricus bisporus* (74.4 mg/g), and* Phellinus linteus* (62.6 mg/g) [13]. The exopolysaccharides extracted from the tested strain showed high content of reducing sugar (61.2 mg/g) (Table 1). The concentration of phenolic compounds in the crude exopolysaccharides was 12 *μ*M and that was almost six times lower than the values obtained for crude extracts of endopolysaccharides from* Cerrena unicolor *[23]. The total protein contents of the crude exopolysaccharide of* G. applanatum* was 22.6 ± 0.7 mg/g dwc (2.2%) (Table 1). Polysaccharide fractions from another strain of the* Ganoderma *genus*, G. lucidum*, also contained proteins (about 6.71%) characterized as glycopeptides [11]. Crude exopolysaccharides isolated from mycelium of* Cordyceps sinensis* described by Leung et al. [30] contained 65–75% of sugar and about 25% of proteins, suggesting their polysaccharide-protein character. Cui and Chisti [31] reported additionally that polysaccharides-peptides complexes from* Coriolus versicolor* contained peptides mainly consisting of aspartic and glutamic acids.

**Figure 1:**
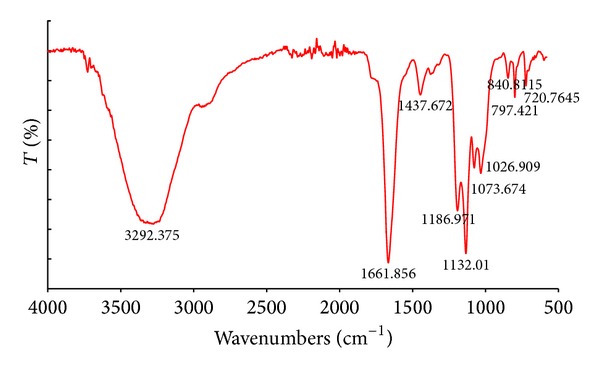
FT-IR spectra of the exopolysaccharides from* G. applanatum* (GpEPS).

**Figure 2:**
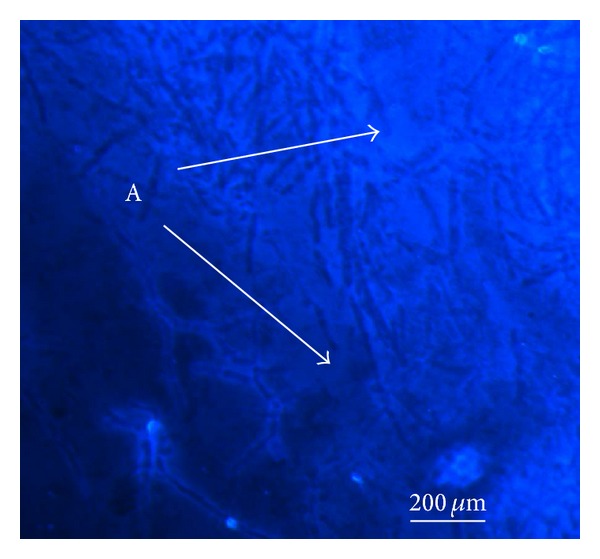
Morphology of exopolysaccharides fibers using confocal laser scanning microscopy. The tested samples of GpEPS were stained with Fluorescence Brightener 28 commonly used in order to detection of *β*-linked polysaccharides. For visualization of the GpEPS, the inverted microscope Axiovert 200 M equipped with an LSM 5 Pascal head (with magnification 200x) was used. The letter (A) indicates the luminous fibers exhibiting visible *β*-linked polysaccharide fragments.

The FT-IR spectrum of the ethanol-extracted exopolysaccharides of* G. applanatum* showed a typical carbohydrate pattern (Figure 1). The absorption band at 3292.3 cm^−1^ indicates the presence of the hydroxyl group (–OH) characteristic for molecular interactions of polysaccharide chains [32]. The two bands towards 1661.6 and 1437.6 cm^−1^ are correlated with the presence of the deprotonated carboxylic group (–COO^−^). The bands at 1026.9, 1132, and 1186 cm^−1^ suggested the presence of C–O bands, and the band at 1073 cm^−1^ was characteristic of the presence of *β*-glucans [33, 34]. Additionally, a characteristic peak at 840.8 cm^−1^ indicating *α*-linked glycosyl residues was observed by Kozarski et al. [13]. In addition, staining of the exopolysaccharides with Fluorescence Brightener 28 confirmed the presence of *β*-linked bonds in the polysaccharides studied (Figure 2). Chemical characterization of the exopolysaccharides properties is presented in Table 1.

### 3.3. Biological Properties of the Crude GpEPS Preparation

#### 3.3.1. Toxic, Antimicrobial, and Antioxidant Properties

It is known that a number of substances isolated from mushrooms may exhibit antibacterial activity. Zhu et al. [35] showed the antibacterial activity of polysaccharides from spent mushroom substrate against* E. coli* and* S. lutea*. Results from our preliminary toxicity tests obtained using the Microtox detection system showed that the exposure of genetically modified marine bacterium* Vibrio fischeri *to tested exopolysaccharides caused 82.8% cell damage. The antibacterial activity of GpEPS was analyzed using* E. coli* and* S. aureus* strains. Exopolysaccharide samples showed antibacterial properties against the* S. aureus* strain with the inhibition zone of 17.9 mm and MIC values 1 mg/mL (Table 2). The results indicate an evident antibacterial effect of the tested preparation.

Polymeric carbohydrates from mushrooms have been reported as modulators of inflammatory response systems. Among others, antioxidant properties of polysaccharides produced by fungi such as* Agaricus bisporus*,* Agaricus brasiliensis*,* Ganoderma lucidum*, and* Phellinus linteus* have been shown [13, 36–38].

The tested GpEPS exhibited relatively weak antioxidant properties and an ability to scavenge free radicals. In the present study, the ability of exopolysaccharide to reduce DPPH was confirmed, but the degree of reduction did not exceed 20% of the antioxidant properties, in comparison to the control. Similar to Kozarski et al. [14], we found that the ability to scavenge free radicals by polymers is related to the presence of large amounts of phenolic compounds. The results obtained may prove the thesis proposed by Kozarski et al. [14] that the low levels of free radical scavenging exhibited by the tested preparation are related to the results of phenolic compounds.

#### 3.3.2. Antitumor Activity

**Figure 3:**
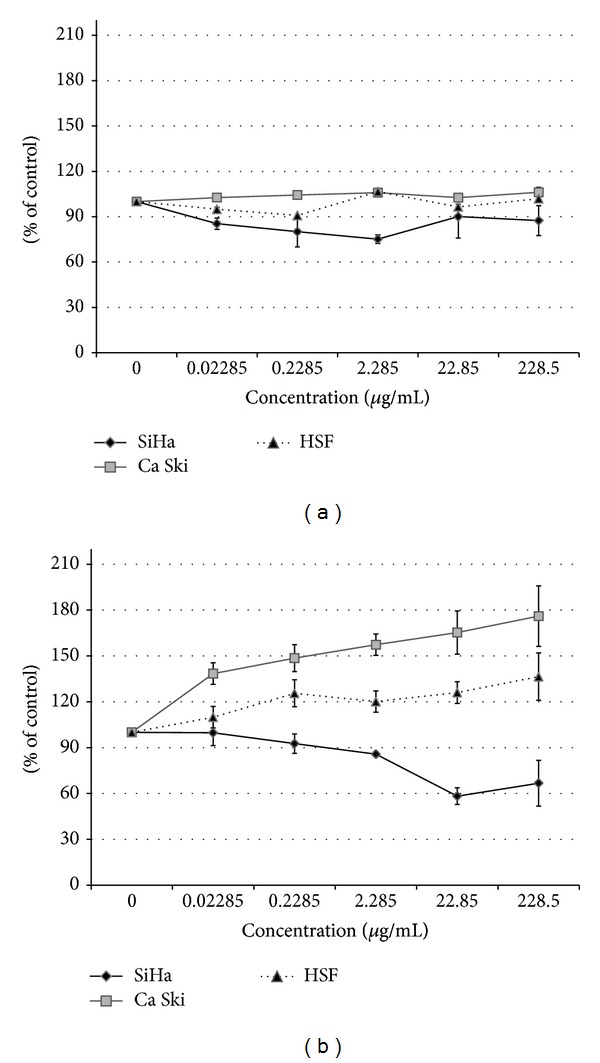
The cytotoxic effect of exopolysaccharides from* G. applanatum *(GpEPS) against carcinoma cell lines (SiHa and Ca Ski) and human skin fibroblast (HSF) after 24 h (a) and 48 h (b) incubation. Each value is expressed as mean ± SD (*n* = 3).

The crude exopolysaccharides extracted from* G. applanatum* were subjected to* in vitro* cytotoxicity assays against carcinoma cell lines (SiHa and Ca Ski) and a human skin fibroblast (HSF) line. After 24 hours of incubation, slight changes in cell viability were observed (HSF, SiHa, Ca Ski), but they were not statistically significant, compared to the control. In contrast, the results obtained after 48 hour incubation were more varied. Our results showed that the isolated polysaccharides exhibited cytotoxic activity against the SiHa carcinoma cell line. A 42.8% and 34% decrease in cell viability at 22.88 *μ*g/mL and 228.5 *μ*g/mL concentrations of the exopolysaccharides studied, respectively, was noted (Figures 3(a) and 3(b)). On the other hand, a ca. 97% and 76% increase in the metabolic activity of Ca Ski cells for the exopolysaccharide concentrations of 22.85 and 228.5 *μ*g/mL, respectively, was observed. In turn, a ca. 10–36% increase in the activity was observed in the case of fibroblasts (HSF). To our knowledge, there are currently no reports on the cytotoxic properties of exopolysaccharides from* G. applanatum*. Li et al. [39] showed that the polysaccharides from* G. atrium* inhibited tumor growth in S180-bearing mice* via *induction of apoptosis through mitochondrial pathways and immunoenhancement effects. Recently, polysaccharide-protein peptide conjugates with anticancer or immunomodulation properties were isolated from* G. lucidium*. For example, a GIPP fraction (polysaccharide-peptide conjugation) was indicated to inhibit proliferation of HUVECs by inducing cell apoptosis and decrease the expression of secreted VEGF in human lung cancer cells [40, 41]. The results of our study indicate that the tested polysaccharide fraction of* G. applanatum* may exhibit selective cytotoxic activity against SiHa cell lines at concentration above 22.85 *μ*g/mL.

**Figure 4:**
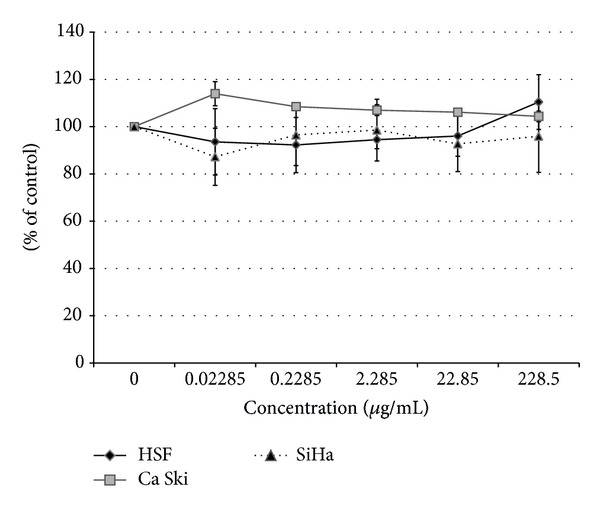
Proliferative activity of carcinoma cell lines (SiHa and Ca Ski) and human skin fibroblast (HSF) in the presence of exopolysaccharides from* G. applanatum *(GpEPS).Each value is expressed as mean ± SD (*n* = 3).

In addition to the cytotoxic activity, antiproliferation activity of exopolysaccharides was also determined. In the presence of the exopolysaccharide from* G. applanatum*, there were no statistically significant changes in cell proliferation activity of the tested cell lines (HSF, SiHA, Ca Ski). The differences in proliferative activity in comparison with the control cells were approximately 4–10% for HSF, 4–13% for Ca Ski, and 2–13% for SiHa cell lines (Figure 4).

#### 3.3.3. Immunomodulatory Activity

**Figure 5:**
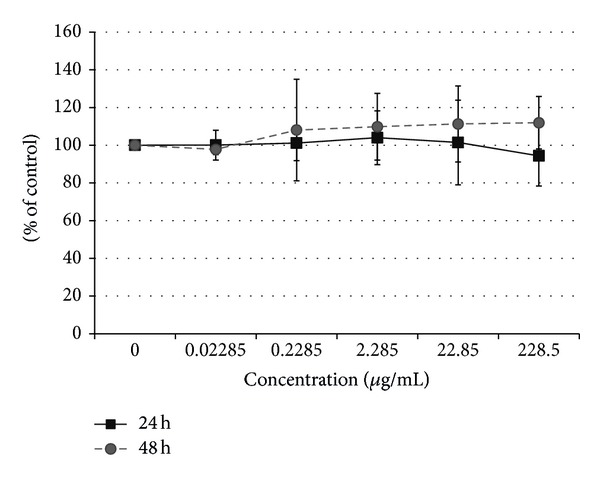
The cytotoxic activity of exopolysaccharides from* G. applanatum* (GpEPS) against macrophages (THP-1). Each value is expressed as mean ± SD (*n* = 3).

**Figure 6:**
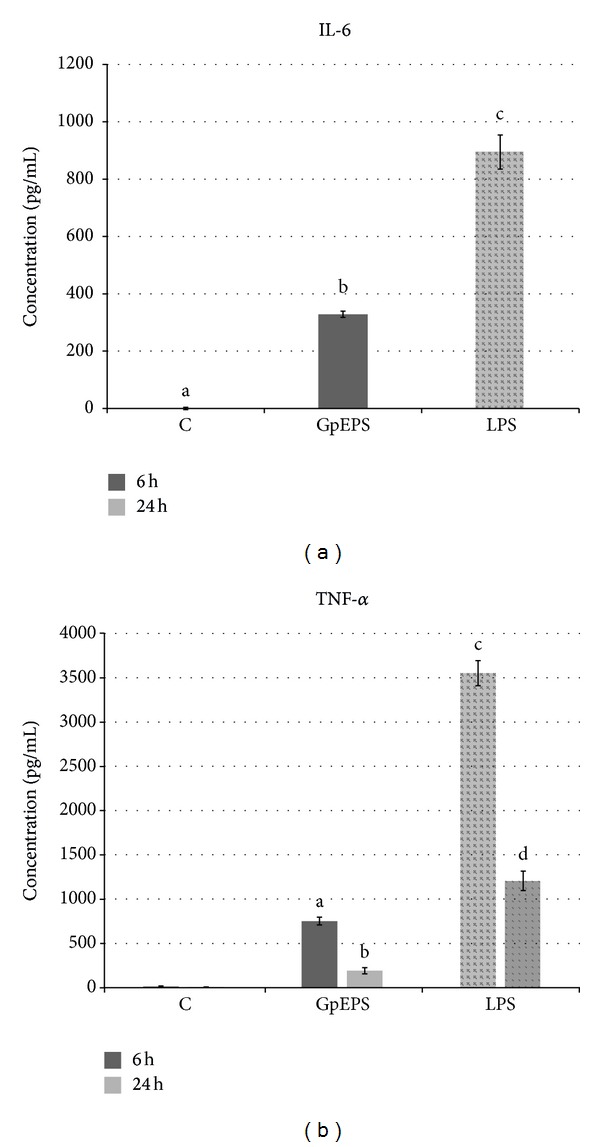
Immunostimulatory activity of exopolysaccharides from* G. applanatum *(GpEPS)*:* (a) the level of IL-6 in the culture fluid after 24 h of treatment with the GpEPS fractions (228.5 *μ*g/mL), (b) the changes of TNF-*α* level after 6 and 24 h of treatment with the GpEPS fractions (228.5 *μ*g/mL) (C-negative control-cultures provided in the RPMI medium with the content of 2% of serum, LPS-positive control, and* E. coli* lipopolysaccharide (10 *μ*g/mL)). All results are expressed as mean ± SD from three experiments (*n* = 3), values marked with the different letters are significantly different (*P* ≤ 0.05).

The isolated from* G. applanatum *exopolysaccharides were tested for their ability to regulate immune response mechanisms. The immunomodulatory properties of the exopolysaccharides were determined by means of THP-1 cells differentiated into macrophages, capable of production of IL-6 and TNF-*α*. A preliminary study of the cytotoxic activity of exopolysaccharides against THP-1-derived macrophages revealed that this fraction was not toxic at all concentrations (Figure 5). Exopolysaccharides at the concentration of 228.5 *μ*g/mL were used for further study of the immunomodulatory activity. The study presented in this paper indicated that after 24 hours of incubation the extracellular polysaccharides from* G. applanatum* stimulated secretion of IL-6 by macrophages at a level of 328.5 pg/mL (Figure 6(a)). Similar results were observed for polysaccharides isolated from* Ganoderma lucidum* which increased the level of proinflammatory cytokines (IL-1*β*, TNF-*α*, and IL-6) secreted by macrophages isolated from rat bone marrow [42]. For comparison, Wang et al. [43] obtained IL-6 at a level of 2933.8 pg/mL in the culture supernatant, incubating peripheral blood mononuclear cells at a density of 1 × 10^6^ cells/mL for 3 days with a fraction of* G. lucidum* polysaccharide at a concentration of 100 mg/mL [43]. Taking into account the fact that Wang et al. [43] used higher cell density and a longer time of incubation, the results (lower level of IL-6) obtained for the fractions examined in this study may not result necessarily from weaker immunomodulatory activity of the tested fractions but rather from the experimental conditions. The 6-hour incubation of extracellular polysaccharides isolated from* G. applanatum* at a concentration of 228.5 *μ*g/mL resulted in production of TNF-*α* by THP-1-derived macrophages at the level of 752.17 pg/mL, representing an approximately 54-fold increase compared to the negative control (Figure 6(b)). In turn, a decrease in the TNF-*α* level to 190.52 pg/mL was observed after 24-hour incubation, although the cytokine level remained higher in comparison with the negative control. Habijanič et al. [44] studied the different polysaccharide fractions from* G. lucidum. *After 4-hour incubation with human peripheral blood mononuclear cells, this polysaccharide (concentration of 200 *μ*g/mL) induced appearance of TNF-*α* at concentrations of approximately 150–600 pg/mL in the culture supernatant. The fungal polysaccharide fractions tested in this study stimulated macrophage production of cytokines at a higher level. Mucopolysaccharides, particularly *β*-glucans, operate as so-called PAMPs, which after nonspecific recognition by the immune system stimulate the immune mechanisms [4].

#### 3.3.4. Testing the Ability of GpEPS to Bind Cholesterol, Triglycerides, Glucose, and Magnesium and Iron Ions

**Figure 7:**
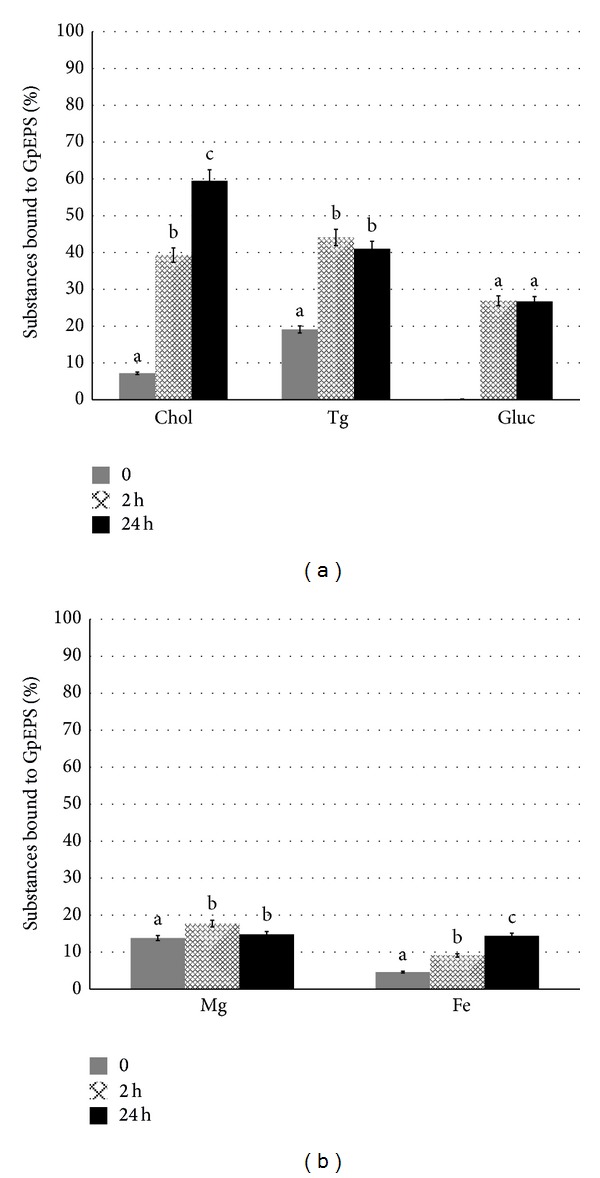
Testing the ability of exopolysaccharides from* G. applanatum *(GpEPS)to binding: cholesterol, triglycerides, glucose, (a) and magnesium and iron ions (b) expressed as a percentage of the test substance bound to exopolysaccharides. All results are expressed as mean ± SD from three experiments (*n* = 3); values marked with the different letters are significantly different (*P* ≤ 0.05).

The ability of fungal polysaccharides to reduce levels of cholesterol and the lipids in blood remains one of the important pharmacological properties. Chen and Huang [45] conducted experiments proving the ability of *β*-glucans to reduce cholesterol levels in blood by partial inhibition of absorption thereof. We confirmed that exopolysaccharides from* G. applanatum* were able to bind* in vitro* cholesterol and triglycerides (Figure 7(a)). The amount of bound cholesterol increased during the incubation time (39.2% after 2 hours and 57.9% after 24 hours of incubation). This correlation was not observed for triglycerides, where the level of bound substances was stable despite the time of incubation (2 hours, 43.3% and 24 hours, 41.6%). The conducted experiments also proved the possibility of glucose binding to the tested GpEPS. The incorporated glucose level was amounted to 26.6% and was independent of the incubation time. Magnesium and iron ions attachment ability (Figure 7(b)) revealed weak capacity of exopolysaccharides to absorption of these substances (17.4% for Mg^2+^ and 14.1% for Fe^2+^). In conclusion, all these data indicate that exopolysaccharides extracted from* G. applanatum* possess a high capability of binding cholesterol and triglycerides; however, further tests* in vivo* are required to confirm their hypocholesterolemic properties.

## 4. Conclusions

The weight of evidence suggests that exopolysaccharides isolated from the white rot fungus* Ganoderma applanatum* are characterized by a lot of important biomedical properties. The conducted experiments have evidently shown an anticancer, immunomodulating, and antibacterial effect. The results of our tests proved that the crude GpEPS preparation exhibited antitumor activity against carcinoma cells (lines SiHa) and stimulated production of Il-6 and TNF-*α* by macrophage line THP-1. At the same time, the antibacterial tests also indicated good antiseptic properties of the exopolysaccharides studied against* S. aureus* and* V. fischeri* strains. The biological properties described as well as the hypocholesterolemic effect of the tested substances suggest that the present studies should be continued in view of future pharmacological applications. However, it is worth noting that further studies comprising preventive and therapeutic actions of the GpEPS fraction are needed. In the promotion of the described preparation as a promising bioactive product, the simple and economical way of production and isolation thereof as well as the possibility of standardization and control of the production conditions should be emphasized.

## Figures and Tables

**Table 1:** Amount of proteins, total polysaccharides, reducing sugar, and total phenolic compounds content of GpEPS. All results are expressed as mean ± SD from three experiments (*n* = 3).

Sample	Extraction yield^a^	Protein contents (mg/g^b^)	Total carbohydrate (mg/g^b^)	Total polysaccharide (mg/g^b^)	Reducing sugar (mg/g^b^)	Total phenolic compounds (*µ*M/g^b^)
GpEPS	8.13 ± 0.4	22.6 ± 0.07	303 ± 1.29	241.8 ± 2	61.2 ± 1.2	12 ± 0.4

^a^g/100 g dry weight basis.

^b^g dry weight of crude exopolysaccharide.

**Table 2:** The antibacterial activities and the toxicity effect of GpEPS (1 mg/mL) isolated from *G. applanatum* submerged cultures. All results are expressed as mean ± SD from three experiments (*n* = 3).

Sample	Diameters of inhibition zone (mm)		Toxic effect (%)
	*E. coli *	*S. aureus *	*V. fischeri *
GpEPS	^ a^—	17.98 ± 0.4	82.6 ± 2.4

^a^Not detected.

## References

[1] Paterson RRM (2006). *Ganoderma*—a therapeutic fungal biofactory. *Phytochemistry*.

[2] Leskosek-Cukalovic I, Despotovic S, Lakic N, Niksic M, Nedovic V, Tesevic V (2010). *Ganoderma lucidum*—medical mushroom as a raw material for beer with enhanced functional properties. *Food Research International*.

[3] Methacanon P, Madla S, Kirtikara K, Prasitsil M (2005). Structural elucidation of bioactive fungi-derived polymers. *Carbohydrate Polymers*.

[4] Chen J, Seviour R (2007). Medicinal importance of fungal *β*-(1→3), (1→6)-glucans. *Mycological Research*.

[5] Lee JS, Kwon JS, Yun JS (2010). Structural characterization of immunostimulating polysaccharide from cultured mycelia of *Cordyceps militaris*. *Carbohydrate Polymers*.

[6] Lindequist U, J Niedermeyer TH, Julich WD (2005). *The Pharmacological Potential of Mushrooms*.

[7] Liu J, Gunn L, Hansen R, Yan J (2009). Combined yeast-derived *β*-glucan with anti-tumor monoclonal antibody for cancer immunotherapy. *Experimental and Molecular Pathology*.

[8] Brown GD, Gordon S (2005). Immune recognition of fungal *β*-glucans. *Cellular Microbiology*.

[9] Shenbhagaraman R, Jagadish LK, Premalatha K, Kaviyarasan V (2012). Optimization of extracellular glucan production from *Pleurotus eryngii* and its impact on angiogenesis. *International Journal of Biological Macromolecules*.

[10] Ooi VEC, Liu F (2000). Immunomodulation and anti-cancer activity of polysaccharide-protein complexes. *Current Medicinal Chemistry*.

[11] Jia J, Zhang X, Hu Y (2009). Evaluation of in vivo antioxidant activities of *Ganoderma lucidum* polysaccharides in STZ-diabetic rats. *Food Chemistry*.

[12] Saltarelli R, Ceccaroli P, Iotti M (2009). Biochemical characterisation and antioxidant activity of mycelium of *Ganoderma lucidum* from Central Italy. *Food Chemistry*.

[13] Kozarski M, Klaus A, Niksic M, Jakovljevic D, Helsper JPFG, van Griensven LJLD (2011). Antioxidative and immunomodulating activities of polysaccharide extracts of the medicinal mushrooms *Agaricus bisporus*, *Agaricus brasiliensis*, *Ganoderma lucidum* and *Phellinus linteus*. *Food Chemistry*.

[14] Kozarski M, Klaus A, Nikšić M (2012). Antioxidative activities and chemical characterization of polysaccharide extracts from the widely used mushrooms *Ganoderma applanatum*, *Ganoderma lucidum*, *Lentinus edodes* and *Trametes versicolor*. *Journal of Food Composition and Analysis*.

[15] Hirasawa M, Shouji N, Neta T, Fukushima K, Takada K (1999). Three kinds of antibacterial substances from *Lentinus edodes* (Berk.) Sing. (Shiitake, an edible mushroom). *International Journal of Antimicrobial Agents*.

[16] Lindeberg G, Holm G (1952). Occurrence of tyrosinase and laccase in fruit bodies andmycelia of some *Hymenomycetes*. *Physiology Plant*.

[17] Borges MJ, Azevedo MO, Bonatelli JR, Felipe MSS, Astolfi-Filho S (1990). A practical method for the preparation of total DNA from filamentous fungi. *Fungal Genetics Newsletters*.

[18] White TJ, Bruns T, Lee S (1990). *PCR Protocols: A Guide to Methods and Applications*.

[19] Dubois M, Gilles KA, Hamilton JK, Rebers PA, Smith F (1956). Colorimetric method for determination of sugars and related substances. *Analytical Chemistry*.

[20] Hope CFA, Burns RG (1987). Activity, origins and location of cellulases in a silt loam soil. *Biology and Fertility of Soils*.

[21] Bradford MM (1976). A rapid and sensitive method for the quantitation of microgram quantities of protein utilizing the principle of protein dye binding. *Analytical Biochemistry*.

[22] Malarczyk E (1998). Transformation of phenolic acids by *Nocardia*. *Acta Microbiologica Polonica*.

[23] Jaszek M, Osińska-Jaroszuk M, Janusz G (2013). New bioactive fungal molecules with high antioxidant and antimicrobial capacity isolated from cerrena unicolor idiophasic cultures. *BioMed Research International*.

[24] Paduch R, Matysik G, Wójciak-Kosior M (2008). *Lamium Album* extracts express free radical scavenging and cytotoxic activities. *Polish Journal of Environmental Studies*.

[25] Usui T, Iwasaki Y, Mizuno T, Tanaka M, Shinkai K, Arakawa M (1983). Isolation and characterization of antitumor active *β*-d-glucans from the fruit bodies of *Ganoderma applanatum*. *Carbohydrate Research*.

[26] Lee WY, Park Y, Ahn JK, Ka KH, Park SY (2007). Factors influencing the production of endopolysaccharide and exopolysaccharide from *Ganoderma applanatum*. *Enzyme and Microbial Technology*.

[27] Manasseh AT, Godwin JTA, Emanghe EU, Borisde OO (2012). Phytochemical properties of *Ganoderma applanatum* as potential agents in the application of nanotechnology in modern day medical practice. *Asian Pacific Journal of Tropical Biomedicine*.

[28] Zhao L, Dong Y, Chen G, Hu Q (2010). Extraction, purification, characterization and antitumor activity of polysaccharides from *Ganoderma lucidum*. *Carbohydrate Polymers*.

[29] Telles CBS, Sabry DA, Almeida-Lima J (2011). Sulfation of the extracellular polysaccharide produced by the edible mushroom *Pleurotus sajor-caju* alters its antioxidant, anticoagulant and antiproliferative properties *in vitro*. *Carbohydrate Polymers*.

[30] Leung PH, Zhao S, Ho KP, Wu JY (2009). Chemical properties and antioxidant activity of exopolysaccharides from mycelial culture of *Cordyceps sinensis* fungus Cs-HK1. *Food Chemistry*.

[31] Cui J, Chisti Y (2003). Polysaccharopeptides of *Coriolus versicolor*: physiological activity, uses, and production. *Biotechnology Advances*.

[32] Carey PR (1992). *Biochemical Application of Raman and Resonance Raman Spectroscopies*.

[33] Stone B, Clarke A (1992). *Chemistry and Biology of (1, 3)-*β*-Glucans*.

[34] Šandula J, Kogan G, Kačuráková M, Machová E (1999). Microbial (1 → 3)—D-glucans, their preparation, physic-chemical characterization and immunomodulatory activity. *Carbohydrate Polymers*.

[35] Zhu H, Sheng K, Yan E, Qiao J, Lv F (2012). Extraction, purification and antibacterial activities of a polysaccharide from spent mushroom substrate. *International Journal of Biological Macromolecules*.

[36] Kao P, Wang S, Hung W, Liao Y, Lin C, Yang W (2012). Structural characterization and antioxidative activity of low-molecular-weights beta-1,3-glucan from the residue of extracted *Ganoderma lucidum* fruiting bodies. *Journal of Biomedicine and Biotechnology*.

[37] Yuan B, Zhang W, Yu Z, Zhang R (2012). *In vitro* evaluation of antioxidant property of the exopolysaccharides peptides from *Ganoderma lucidum* CAU5501 in submerged culture. *Journal of Food, Agriculture and Environment*.

[38] Zhu X, Chen X, Xie J, Wang P, Su W (2012). Mechanochemical-assisted extraction and antioxidant activity of polysaccharides from *Ganoderma lucidum* spores. *International Journal of Food Science & Technology*.

[39] Li WJ, Chen Y, Nie SP (2011). *Ganoderma atrum* polysaccharide induces anti-tumor activity via the mitochondrial apoptotic pathway related to activation of host immune response. *Journal of Cellular Biochemistry*.

[40] Cao QZ, Lin ZB (2006). *Ganoderma lucidum* polysaccharides peptide inhibits the growth of vascular endothelial cell and the induction of VEGF in human lung cancer cell. *Life Sciences*.

[41] Zong A, Cao H, Wang F (2012). Anticancer polysaccharides from natural resources: a review of recent research. *Carbohydrate Polymers*.

[42] Lull C, Wichers HJ, Savelkoul HFJ (2005). Antiinflammatory and immunomodulating properties of fungal metabolites. *Mediators of Inflammation*.

[43] Wang SY, Hsu HC, Tzeng CH, Lee SS, Shiao MS, Ho CK (1997). The anti-tumor effect of *Ganoderma lucidum* is mediated by cytokines released from activated macrophages and T lymphocytes. *International Journal of Cancer*.

[44] Habijanič J, Berovič M, Wraber B, Hodzar D, Boh B (2001). Immunostimulatory effects of polysaccharides from *Ganoderma lucidum* submerged biomass cultivation. *Food Technology and Biotechnology*.

[45] Chen J, Huang X (2009). The effects of diets enriched in beta-glucans on blood lipoprotein concentrations. *Journal of Clinical Lipidology*.

